# Use and Control of Artificial Intelligence in Patients Across the Medical Workflow: Single-Center Questionnaire Study of Patient Perspectives

**DOI:** 10.2196/24221

**Published:** 2021-02-17

**Authors:** Simon Lennartz, Thomas Dratsch, David Zopfs, Thorsten Persigehl, David Maintz, Nils Große Hokamp, Daniel Pinto dos Santos

**Affiliations:** 1 Institute for Diagnostic and Interventional Radiology Faculty of Medicine and University Hospital Cologne University of Cologne Cologne Germany

**Keywords:** artificial intelligence, clinical implementation, questionnaire, survey

## Abstract

**Background:**

Artificial intelligence (AI) is gaining increasing importance in many medical specialties, yet data on patients’ opinions on the use of AI in medicine are scarce.

**Objective:**

This study aimed to investigate patients’ opinions on the use of AI in different aspects of the medical workflow and the level of control and supervision under which they would deem the application of AI in medicine acceptable.

**Methods:**

Patients scheduled for computed tomography or magnetic resonance imaging voluntarily participated in an anonymized questionnaire between February 10, 2020, and May 24, 2020. Patient information, confidence in physicians vs AI in different clinical tasks, opinions on the control of AI, preference in cases of disagreement between AI and physicians, and acceptance of the use of AI for diagnosing and treating diseases of different severity were recorded.

**Results:**

In total, 229 patients participated. Patients favored physicians over AI for all clinical tasks except for treatment planning based on current scientific evidence. In case of disagreement between physicians and AI regarding diagnosis and treatment planning, most patients preferred the physician’s opinion to AI (96.2% [153/159] vs 3.8% [6/159] and 94.8% [146/154] vs 5.2% [8/154], respectively; *P*=.001). AI supervised by a physician was considered more acceptable than AI without physician supervision at diagnosis (confidence rating 3.90 [SD 1.20] vs 1.64 [SD 1.03], respectively; *P*=.001) and therapy (3.77 [SD 1.18] vs 1.57 [SD 0.96], respectively; *P*=.001).

**Conclusions:**

Patients favored physicians over AI in most clinical tasks and strongly preferred an application of AI with physician supervision. However, patients acknowledged that AI could help physicians integrate the most recent scientific evidence into medical care. Application of AI in medicine should be disclosed and controlled to protect patient interests and meet ethical standards.

## Introduction

The incremental use of artificial intelligence (AI) is widely considered as one of the most disruptive developments of the past decades [1]. In medicine, increasing evidence has revealed the promising applications of AI for disease prevention, diagnosis, and treatment [2-4]. Among these, specialties relying on the interpretation of medical imaging data, such as dermatology [5], pathology [6], and radiology [7-9], have a particular scientific and economic focus.

While several studies suggested that AI might outperform board-certified physicians at narrow diagnostic tasks [10-13], broad clinical implementation of such technologies has not matched the pace of scientific advancements. Among practical reasons, such as high heterogeneity in clinical data and clinical workflows as well as cost efficiency considerations affecting clinical implementation, other concerns pertain to ethical questions and liability. Consequently, different concepts of AI implementation in existing clinical workflows in a controlled and responsible manner have been discussed to preserve pivotal pillars of accountability [14]. In this regard, one important consensus is that AI should remain as transparent and explainable as possible.

To implement AI in an acceptable manner, it is important to understand perspectives on AI use in clinical routines from among stakeholders, including patients and health care professionals such as students, physicians, and caregivers [15]. In this respect, most studies have reported that medical professionals in specialties most evidently influenced by AI agree with its implementation and consider it a tool complementing the armamentarium they regularly work with. Despite dire early predictions—for example, radiologists being potentially replaced by AI [16]—recent studies have demonstrated a more gradual adaptation of AI solutions that currently augment human capabilities rather than replacing them [17].

Regarding patients’ perspectives on the use of AI in medicine, current studies are primarily focused on particular subspecialties or individual diagnostic procedures [18-20]. Although most of these studies indicate that patients generally accept the implementation of AI in medicine, a recent study investigating patients’ perspectives on implementing specific AI devices revealed controversial opinions among numerous patients, particularly regarding the question of human control [21].

Therefore, this study aimed to investigate patients’ perspectives on the clinical implementation of AI in a more coherent approach that includes the assessment of key clinical competencies such as physician–patient interaction, diagnosis, and therapy as integral parts of the medical workflow. Further, we investigated opinions for human control of AI and its acceptance depending on different disease severities.

## Methods

### Participants

After our single-center survey study was approved by the institutional review board (approval number 19-1552), patients scheduled for cross-sectional imaging between February 10, 2020, and May 24, 2020, were informed about the possibility to voluntarily complete an anonymous questionnaire on registration. Dedicated boxes for returning the completed questionnaires were placed in the waiting areas. Questionnaires were collected and data were manually transferred to a structured spreadsheet (Excel, Microsoft Corp) at the end of the acquisition period. The response rate was calculated as proposed by the American Association for Public Opinion Research, using the following formula:

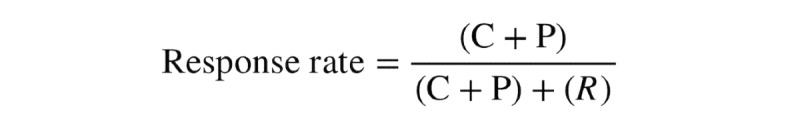



where C is the number of completed questionnaires; P, the number of partially completed questionnaires; and R, the number of nonresponders not consenting to participate or opting out of the questionnaire study.

### Questionnaire

A senior expert in medical AI, a radiology resident with 4 years of experience and a PhD in psychology, conducted a literature review on previous surveys on AI in general and in medicine. As previous surveys were focused on particular subspecialties or limited in their scope, a new survey was generated. Patient interaction, diagnostics, and treatment decisions were identified as key clinical competencies and were therefore implemented as central elements to assess patients’ acceptance towards the application of AI in medicine. Because no previous questionnaire comprising all the specific endpoints of our study was available, external validation was omitted. The questionnaire comprised five subsections. The first subsection inquired age, gender, level of education, and a history of a cancer diagnosis. Moreover, prior knowledge of AI had to be indicated. In the second subsection, participants were asked about their confidence in physicians versus AI in different clinical tasks, including the assessment of the medical history of a patient, making of diagnostic and treatment decisions, and addressing of the patients’ fears and need for information. The third subsection determined patients’ opinions on human control of AI at diagnosis and treatment planning. In the fourth subsection, the respondents were asked to state whose decision should be preferred at diagnosis and treatment planning in case of disagreement between the physician and AI. Finally, in the fifth subsection, participants were asked to indicate their acceptance regarding the application of AI in diagnosing and treating diseases of different severity. In the second, third, and fifth subsections, patients were asked to indicate their agreement based on a Likert scale ranging from 1 (“I strongly disagree”) to 5 (“I strongly agree”). In subsection 4, a binary decision between AI and physician was requested, with the option to choose “I don’t know/I don’t have an opinion on this.”

### Sample Size Estimation

A power analysis was performed for sample size estimation. In order not to disregard smaller, yet important differences in this new research field, our study was sufficiently powered to detect small effects (Cohen *d*=0.2). With α=.05 and power=0.80, the projected sample size needed to detect a small effect (Cohen *d*=0.2) for within-group comparisons was N=199 [22]. Therefore, the inclusion of at least 200 participants was deemed necessary.

### Statistical Analysis

Data were analyzed using SPSS (version 25, IBM Corp) as well as R 3.4.0 with RStudio 1.0.136 [23]. Likert scores were compared using two-tailed, paired samples *t* tests. Chi-squared tests were used to compare the proportions of participants. Pearson correlation analysis was used to assess the association between prior knowledge of AI and confidence on physicians and AI. A *P* value below .05 was considered statistically significant.

## Results

### Participants

Questionnaire outcomes obtained from 229 patients (99 male, 112 female, 18 of unspecified gender; age 18-82 years) scheduled for computed tomography (CT) or magnetic resonance imaging (MRI) were included. The demographic characteristics, education, prior knowledge of AI and the history of cancer diagnosis of the questionnaire participants are summarized in Table 1. In total, 515 questionnaires were handed out, of which 229 were completed and 19 were incomplete (response rate 48.2%).

**Table 1:** Data obtained from the questionnaire participants (N=229).

Characteristic		Participants
Age (years), mean (SD)		51.8 (15.4)
**Gender, n (%)**		
	Men	99 (43.2)
	Women	112 (48.9)
	Nonbinary	0 (0)
	Not indicated	18 (7.9)
**Level of education, n (%)**		
	1 (ISCED^a^ 1-2)	57 (24.9)
	2 (ISCED 3-5)	71 (31.0)
	3 (ISCED 6-8)	84 (36.7)
	Not indicated	17 (7.4)
**Prior knowledge of artificial intelligence (1=completely unfamiliar; 5=very familiar), n (%)**		
	1	39 (17)
	2	46 (20.1)
	3	79 (34.5)
	4	28 (12.2)
	5	8 (3.5)
	Not indicated	29 (12.7)
**History of oncologic disease, n (%)**		
	Previous cancer diagnosis	144 (62.9)
	No previous cancer diagnosis	66 (28.8)
	Not indicated	19 (8.3)

^a^ISCED: International Standard Classification of Education. ISCED levels: 1=primary education, 2=lower secondary education, 3=upper secondary education, 4=postsecondary nontertiary education, 5=short-cycle tertiary education, 6=bachelor or equivalent, 7=master or equivalent, 8=doctoral or equivalent.

### Confidence in the Capabilities of Physicians vs AI

**Figure 1:**
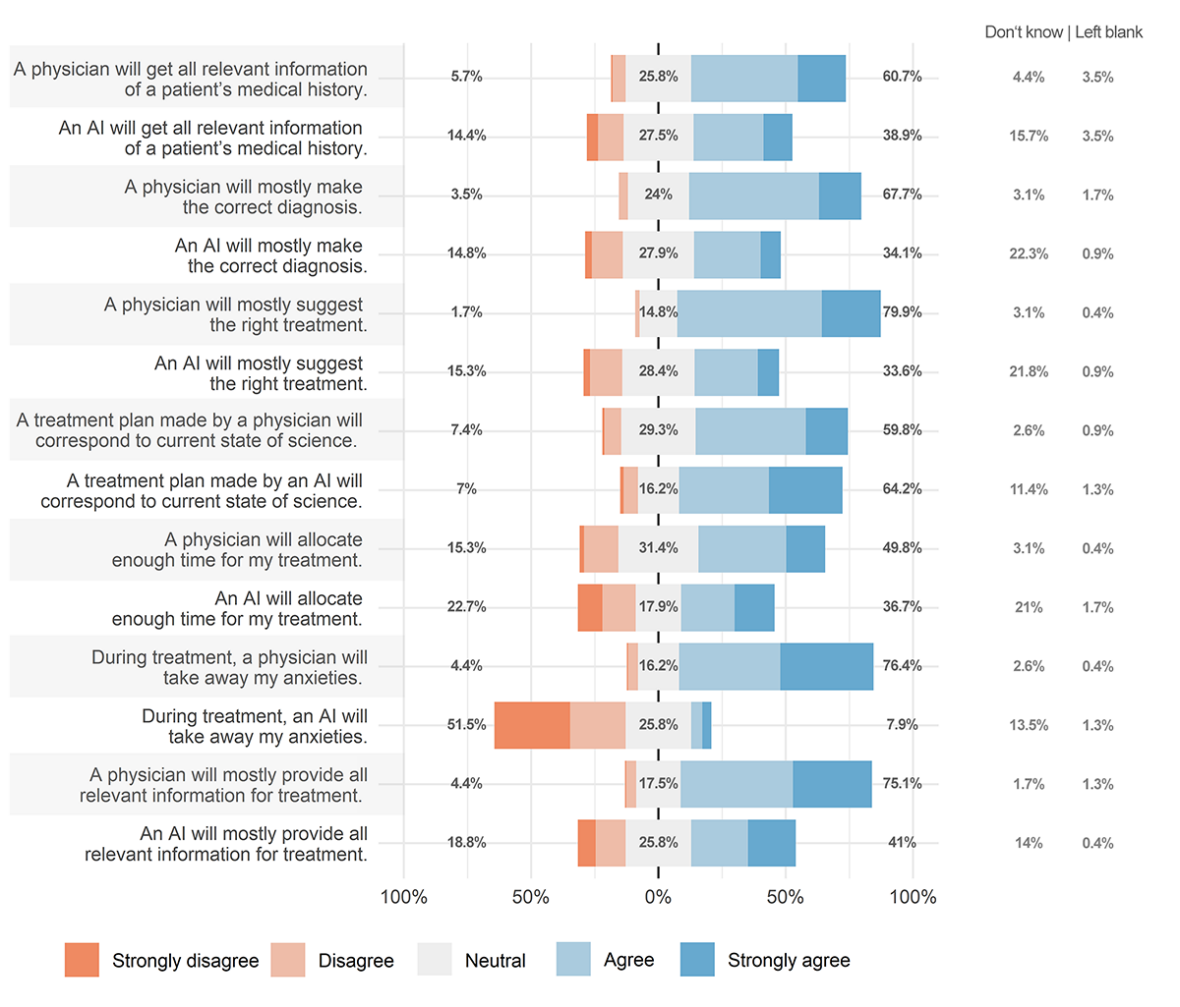
Results of the questionnaire regarding the clinical capabilities of physicians versus artificial intelligence (AI). Percentages refer to the proportion of negative (light orange, orange), neutral (gray), and positive (light blue, blue) responses. Proportions of patients who indicated “Don’t know” or left the question blank are indicated on the right. Patients favored physicians to AI for all clinical capabilities except for making a treatment plan based on current clinical knowledge, where they preferred an AI algorithm.

Patients assigned significantly higher mean scores to the physician rather than to AI for all capabilities included (Table 2), except for treatment planning based on the most recent scientific evidence, for which the participants favored AI to physicians (3.96 [SD 0.95] vs 3.71 [SD 0.84]; mean difference –0.255; 95% CI –0.416 to –0.094; t_195_=–3.12; *P*=.002, Cohen *d*=–0.233). Figure 1 summarizes the proportions of ratings assigned to physicians and AI for different clinical tasks.

**Table 2:** Comparison of mean ratings regarding confidence in clinical capabilities of physicians and artificial intelligence (AI).

Capability	Physician, mean (SD)	AI, mean (SD)	Mean difference (95% CI)	*t* (*df*)	*P* value	Cohen *d*
Obtaining any relevant information from my medical history	3.81 (0.85)	3.38 (1.04)	0.433 (0.258 to 0.608)	4.88 (177)	<.001	0.407
Making an accurate diagnosis	3.91 (0.76)	3.27 (0.96)	0.633 (0.454 to 0.812)	6.99 (168)	<.001	0.617
Proposing the appropriate treatment	4.08 (0.64)	3.30 (0.98)	0.78 (0.621 to 0.94)	9.67 (172)	<.001	0.959
Planning treatment according to recent state of science	3.71 (0.84)	3.96 (0.95)	–0.255 (–0.416 to –0.094)	–3.12 (195)	.002	0.233
Allocating a sufficient amount of time for me	3.55 (0.99)	3.25 (1.30)	0.295 (0.043 to 0.547)	2.31 (172)	.02	0.208
Taking away my worries and addressing my anxieties	4.15 (0.86)	2.16 (1.08)	1.984 (1.79 to 2.179)	20.16 (192)	<.001	1.653
Providing all information relevant to my treatment	4.08 (0.80)	3.38 (1.20)	0.699 (0.488 to 0.911)	6.52 (192)	<.001	0.598

### Different Levels of Human Control of AI at Diagnosis and Treatment Planning

During both diagnosis and treatment, patients were significantly more comfortable with the use of AI under the physician’s supervision than without such supervision (at diagnosis: 3.90 [SD 1.20] vs 1.64 [SD 1.03]; mean difference 2.26; 95% CI 2.08 to 2.43; t_213_=25.19; *P*=.001; Cohen *d*=1.62; treatment planning: 3.77 [SD 1.18] vs 1.57 [SD 0.96]; mean difference=2.20; 95% CI 2.03 to 2.38; t_209_=25.12; *P*=.001; Cohen *d*=1.58).

### Disagreement Between Physicians and AI

When asked whose decision should be followed in case of disagreement between the physician and AI regarding the diagnosis, 66.8% (153/229) of patients decided that the diagnosis of the physician should be followed, 2.6% (n=6) of patients decided that the decision of the AI should be followed, 24.5% (n=56) of patients responded that they were undecided, and 6.1% (n=14) of patients did not respond to the question. When analyzing the responses of patients who decided on either AI or a physician, a significantly larger proportion of patients (153/159, 96.2%) decided that the diagnosis of the physician should be considered (*χ*²_1_=135.91; *P*=.001). The same applied to disagreement regarding treatment decisions, for which a similarly large proportion of participants (146/154, 94.8%) decided that the treatment suggested by the physician should be considered (*χ*²_1_=123.66; *P=*.001).

### Application of AI for the Diagnosis and Treatment of Diseases of Different Severity

**Figure 2:**
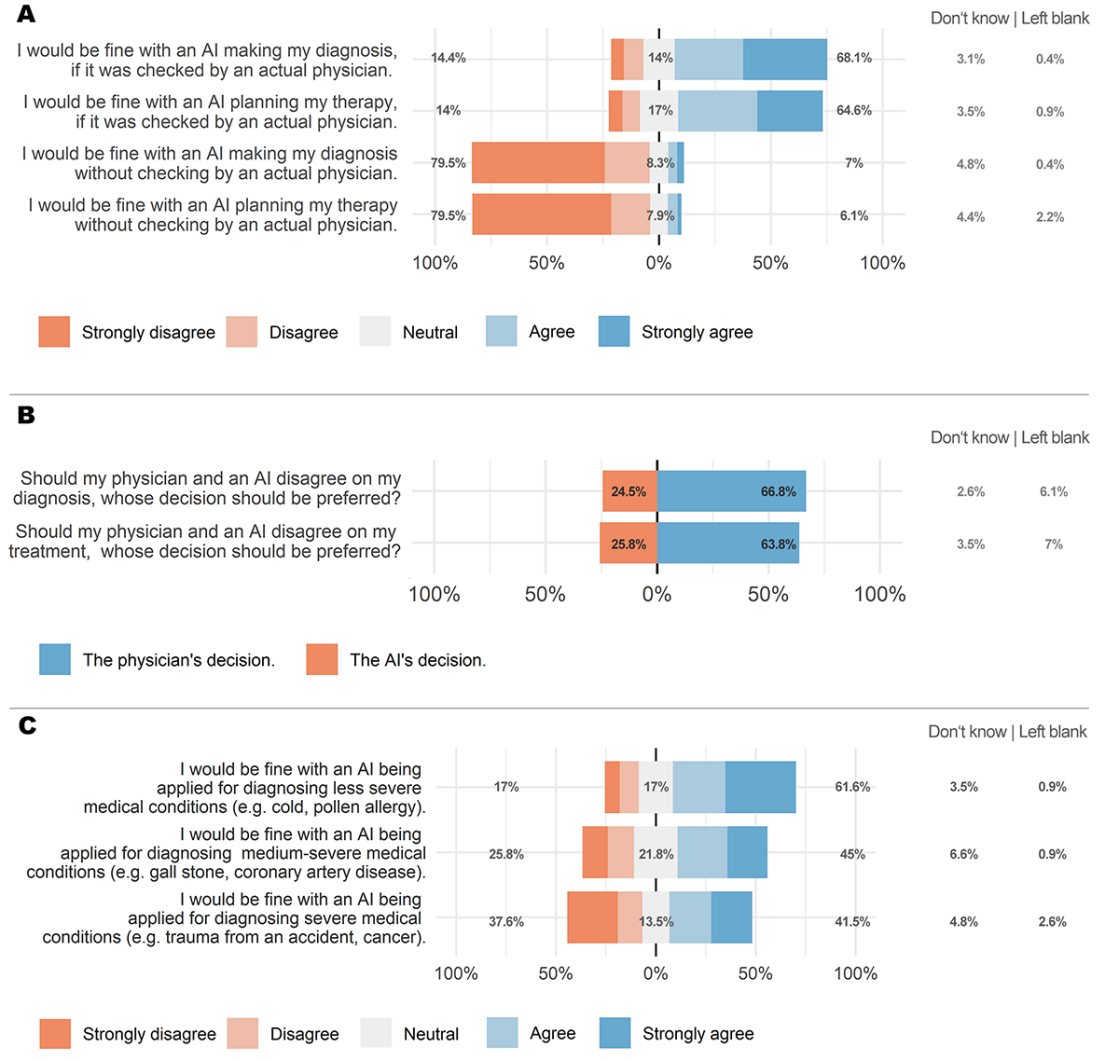
Results of the questionnaire regarding control of artificial intelligence (AI) (A), disagreement between AI and physicians (B), and the application of AI for diagnosing medical conditions of low, medium, and high severity (C). Percentages refer to the proportion of negative (light orange, orange), neutral (gray), and positive (light blue, blue) responses. Proportions of patients who indicated “Don’t know” or left the question blank are indicated on the right.

There was a significant main effect for disease severity (*F*_2,414_=51.75; *P*=.001; η^2^_p_=0.200), indicating that the acceptance of AI was lower for more severe diseases than for less severe diseases. Post hoc *t* tests revealed that the acceptance of AI was significantly lower for diseases of medium severity (3.29 [SD 1.32]) than for those of low severity (3.77 [SD 1.27]; mean difference=0.48; 95% CI 0.33 to 0.64; t_211_=6.15; *P*=.001; Cohen *d*=0.426). Additionally, the acceptance of AI was significantly lower for diseases of high severity (2.97 [SD 1.52]) than for diseases of medium severity (3.30 [SD 1.33]; mean difference=0.33; 95% CI 0.23 to 0.43; t_207_=6.42; *P*=.001; Cohen *d*=0.498). Figure 2 provides an overview of the proportions of ratings regarding human control of AI, disagreement between AI and physicians, and acceptance of AI for the diagnosis and treatment of diseases of different severity.

### Correlation Between Patient-Related Factors and Responses

Prior knowledge of AI was by far the most important patient-related factor correlating with certain patients’ opinions on AI. As shown in Table 3, prior knowledge of AI was significantly correlated with the acceptance of AI in almost all questionnaire items, indicating that patients assigning a higher rating to their prior knowledge on AI were generally more accepting of the use of AI for various aspects of medical treatment and diagnosis. However, the strength of the correlation was weak overall.

**Table 3:** Correlation between prior knowledge of artificial intelligence (AI) and assigned ratings for clinical capabilities of physicians and AI.

Criteria	Correlation between prior knowledge of AI and assigned ratings to physicians			Correlation between prior knowledge of AI and assigned ratings to AI	
	*r*	*P* value	*r*		*P* value
Physician/AI would be capable of obtaining any relevant information from my medical history	–0.015	.83	.193		.01
Physician/AI would be capable of making an accurate diagnosis	0.112	.12	0.170		.03
Physician/AI would be capable of proposing the appropriate treatment	0.103	.16	0.213		.007
Physician/AI would be capable of planning my treatment according to recent state of science	0.062	.39	0.295		<.001
Physician/AI would be capable of allocating a sufficient amount of time for me	0.012	.87	0.269		<.001
Physician/AI would be capable of taking away my worries and addressing my anxieties	–0.001	.99	0.310		<.001
Physician/AI would be capable of providing all information relevant to my treatment	0.016	.83	0.206		.007

## Discussion

Over the past few years, research and development in AI has gained considerable momentum [24]. Although AI in medicine is faced with critical challenges regarding its implementation and reimbursement [25], AI technologies will increasingly impact the diagnosis, management, and treatment of diseases in the future.

This study shows that patients would trust physicians over AI in most clinical capabilities except for basing treatment decisions on the most current clinical knowledge, for which AI was considered superior. This is an interesting finding, as it may be increasingly difficult for physicians both in academia and private practice to keep up with the rapidly growing literature in their corresponding subspecialties [26]. Our results indicate that patients seem to be aware of this issue and consider AI superior in incorporating the most recent scientific evidence. It is worth noting that the discrepancy between the acceptance of AI and physicians was largest in the category of “taking away my worries and addressing my anxieties,” for which physicians received the highest ratings. While this is, to an extent, an expected result, it clearly underlines the demand for empathetic doctor-patient interactions, concurrent with previous findings [27].

Interestingly, patients favored the capabilities of physicians to AI for diagnosis and treatment decisions, although these two aspects have received extensive media coverage, showing promise in its application in medicine. However, it is important to note that although physicians received higher ratings, a large proportion of patients still had positive views on the clinical capabilities of AI. This “cautious optimism” regarding the usefulness of AI in medicine was reflected by the broad consensus among patients for the use of AI for diagnosing and treating rather mild medical conditions; however, this optimism significantly declined with an increase in disease severity. This is in line with a previous survey outlining that acceptance of AI-based decisions declines when the stakes or risks are higher [28]. This observation is relevant in view of many commercially available algorithms for diagnosing life-threatening disease conditions such as cerebral hemorrhage, pulmonary embolism, or pneumothorax [29-31].

Because we are in the era of “narrow AI,” in which algorithms can fulfil very specific tasks with high accuracy [32], the general conception of AI implementation is to use it as a tool to support clinicians in specific areas. However, it can be expected that with the development of AI in medicine, some tools might offer broader and more general applications. Different models of implementation of AI in clinical workflows have been conceived, in which the level of autonomy assigned to AI algorithms plays an important role. In this study, most patients reported that AI findings should be double-checked by a physician. In case of a disagreement between physicians and AI, the vast majority of patients (96.2% [153/159] for disagreement on diagnosis and 94.8% [146/154] for disagreement on treatment decisions) preferred the physician’s to that of AI, concurrent with the results of a previous survey among Chinese cancer patients, in which 88.8% and 91.3% of participants reported they would follow the physician’s suggestion regarding diagnosis and treatment [27].

The use of AI in medicine without adequate disclosure or explanation to patients can be hazardous [33,34]. Consequently, transparency and explicability are absolutely crucial for AI implementation [14]. Based on these findings on AI control and a significant trend towards lower acceptance rates of AI with increasing disease severity, we conclude that patients should be informed of which tasks involve AI algorithms and whether these applications are supervised by a physician. This is particularly relevant as most AI tools being developed and made available fall under this severe disease category, which explicitly comprised oncologic diseases in our questionnaire.

Our study has limitations that need to be acknowledged. The number of participants we included was rather small, which limits generalization of our results to other populations; for example, a previous study suggested that Asian populations may anticipate a more disruptive development of medical AI with the potential replacement of health care professionals [35]. Apart from prior knowledge of AI, we did not observe other significant influences on its acceptance in the medical workflow, which might be attributed to the small sample. However, considering previous reports, we speculate that familiarity with AI technology is indeed the most important factor influencing patients’ acceptance of it. Another limitation to consider is that the setting of handing out the questionnaire at registration for cross-sectional imaging, owing to organizational prerequisites, certainly introduced a selection bias towards participants with a history of more severe diseases warranting such radiological examinations. Surveying patients at primary care physician appointments might therefore yield divergent results.

In conclusion, patients had greater confidence in physicians than in AI in most clinical capabilities except for making treatment decisions based on the most recent scientific evidence, where they found AI advantageous. Patients strongly preferred physician-controlled application of AI. In order to safeguard patient interests, disclosure and control of AI application in medicine is crucial.

## Abbreviations

AI: artificial intelligence
CT: computed tomography
MRI: magnetic resonance imaging

## References

[1] Girasa R (2020). AI as a Disruptive Technology. Artif Intell as a Disruptive Technol.

[2] Graffy PM, Sandfort V, Summers RM, Pickhardt PJ (2019). Automated Liver Fat Quantification at Nonenhanced Abdominal CT for Population-based Steatosis Assessment. Radiology.

[3] Hinton G (2018). Deep Learning-A Technology With the Potential to Transform Health Care. JAMA.

[4] Wang C, Zhu X, Hong JC, Zheng D (2019). Artificial Intelligence in Radiotherapy Treatment Planning: Present and Future. Technol Cancer Res Treat.

[5] Esteva A, Kuprel B, Novoa RA, Ko J, Swetter SM, Blau HM, Thrun S (2017). Dermatologist-level classification of skin cancer with deep neural networks. Nature.

[6] Chang HY, Jung CK, Woo JI, Lee S, Cho J, Kim SW, Kwak T (2019). Artificial Intelligence in Pathology. J Pathol Transl Med.

[7] Alexander A, McGill M, Tarasova A, Ferreira C, Zurkiya D (2019). Scanning the Future of Medical Imaging. J Am Coll Radiol.

[8] Hosny A, Parmar C, Quackenbush J, Schwartz LH, Aerts HJWL (2018). Artificial intelligence in radiology. Nat Rev Cancer.

[9] Choy G, Khalilzadeh O, Michalski M, Do S, Samir AE, Pianykh OS, Geis JR, Pandharipande PV, Brink JA, Dreyer KJ (2018). Current Applications and Future Impact of Machine Learning in Radiology. Radiology.

[10] Rodriguez-Ruiz A, Lång Kristina, Gubern-Merida A, Broeders M, Gennaro G, Clauser P, Helbich TH, Chevalier M, Tan T, Mertelmeier T, Wallis MG, Andersson I, Zackrisson S, Mann RM, Sechopoulos I (2019). Stand-Alone Artificial Intelligence for Breast Cancer Detection in Mammography: Comparison With 101 Radiologists. J Natl Cancer Inst.

[11] Ström Peter, Kartasalo K, Olsson H, Solorzano L, Delahunt B, Berney DM, Bostwick DG, Evans AJ, Grignon DJ, Humphrey PA, Iczkowski KA, Kench JG, Kristiansen G, van der Kwast TH, Leite KRM, McKenney JK, Oxley J, Pan C-C, Samaratunga H, Srigley JR, Takahashi H, Tsuzuki T, Varma M, Zhou M, Lindberg J, Lindskog Cecilia, Ruusuvuori P, Wählby Carolina, Grönberg Henrik, Rantalainen M, Egevad L, Eklund M (2020). Artificial intelligence for diagnosis and grading of prostate cancer in biopsies: a population-based, diagnostic study. Lancet Oncol.

[12] McKinney SM, Sieniek M, Godbole V, Godwin J, Antropova N, Ashrafian H, Back T, Chesus M, Corrado GS, Darzi A, Etemadi M, Garcia-Vicente F, Gilbert FJ, Halling-Brown M, Hassabis D, Jansen S, Karthikesalingam A, Kelly CJ, King D, Ledsam JR, Melnick D, Mostofi H, Peng L, Reicher JJ, Romera-Paredes B, Sidebottom R, Suleyman M, Tse D, Young KC, De Fauw J, Shetty S (2020). International evaluation of an AI system for breast cancer screening. Nature.

[13] Ardila D, Kiraly AP, Bharadwaj S, Choi B, Reicher JJ, Peng L, Tse D, Etemadi M, Ye W, Corrado G, Naidich DP, Shetty S (2019). End-to-end lung cancer screening with three-dimensional deep learning on low-dose chest computed tomography. Nat Med.

[14] Geis JR, Brady AP, Wu CC, Spencer J, Ranschaert E, Jaremko JL, Langer SG, Kitts AB, Birch J, Shields WF, van den Hoven van Genderen R, Kotter E, Gichoya JW, Cook TS, Morgan MB, Tang A, Safdar NM, Kohli M (2019). Ethics of Artificial Intelligence in Radiology: Summary of the Joint European and North American Multisociety Statement. J Am Coll Radiol.

[15] Pinto Dos Santos D, Giese D, Brodehl S, Chon SH, Staab W, Kleinert R, Maintz D, Baeßler B (2019). Medical students' attitude towards artificial intelligence: a multicentre survey. Eur Radiol.

[16] European Society of Radiology (ESR) (2019). What the radiologist should know about artificial intelligence - an ESR white paper. Insights Imaging.

[17] Arieno A, Chan A, Destounis SV (2019). A Review of the Role of Augmented Intelligence in Breast Imaging: From Automated Breast Density Assessment to Risk Stratification. AJR Am J Roentgenol.

[18] Nelson CA, Pérez-Chada LM, Creadore A, Li SJ, Lo K, Manjaly P, Pournamdari AB, Tkachenko E, Barbieri JS, Ko JM, Menon AV, Hartman RI, Mostaghimi A (2020). Patient Perspectives on the Use of Artificial Intelligence for Skin Cancer Screening: A Qualitative Study. JAMA Dermatol.

[19] Palmisciano P, Jamjoom AAB, Taylor D, Stoyanov D, Marcus HJ (2020). Attitudes of Patients and Their Relatives Toward Artificial Intelligence in Neurosurgery. World Neurosurg.

[20] Haan M, Ongena YP, Hommes S, Kwee TC, Yakar D (2019). A Qualitative Study to Understand Patient Perspective on the Use of Artificial Intelligence in Radiology. J Am Coll Radiol.

[21] Tran V, Riveros C, Ravaud P (2019). Patients' views of wearable devices and AI in healthcare: findings from the ComPaRe e-cohort. NPJ Digit Med.

[22] Faul F, Erdfelder E, Buchner A, Lang A (2009). Statistical power analyses using G*Power 3.1: tests for correlation and regression analyses. Behav Res Methods.

[23] Allaire J (2012). RStudio: integrated development environment for R. RStudio.

[24] Pesapane F, Codari M, Sardanelli F (2018). Artificial intelligence in medical imaging: threat or opportunity? Radiologists again at the forefront of innovation in medicine. Eur Radiol Exp.

[25] Banja J (2020). AI Hype and Radiology: A Plea for Realism and Accuracy. Radiology: Artificial Intelligence.

[26] Fontelo P, Liu F (2018). A review of recent publication trends from top publishing countries. Syst Rev.

[27] Yang K, Zeng Z, Peng H, Jiang Y (2019). Attitudes Of Chinese Cancer Patients Toward The Clinical Use Of Artificial Intelligence. Patient Prefer Adherence.

[28] Smith A (2018). Public Attitudes Toward Computer Algorithms. Pew Research Center Internet & Technology.

[29] Kuo W, Hӓne C, Mukherjee P, Malik J, Yuh EL (2019). Expert-level detection of acute intracranial hemorrhage on head computed tomography using deep learning. Proc Natl Acad Sci U S A.

[30] Truong K (2019). Aidoc gets FDA nod for AI pulmonary embolism screening tool. MedCity News.

[31] Shefayim K (2019). Zebra Medical Vision Receives FDA Approval for World’s First AI Chest X-ray Triage Product. Zebra Medical Vision.

[32] Davenport T, Kalakota R (2019). The potential for artificial intelligence in healthcare. Future Healthc J.

[33] Cohen IG (2020). Informed Consent and Medical Artificial Intelligence: What to Tell the Patient?. SSRN Journal.

[34] Robins R, Brodwin E (2020). An invisible hand: Patients aren?t being told about the AI systems advising their care Internet. 2020.

[35] Gao S, He L, Chen Y, Li D, Lai K (2020). Public Perception of Artificial Intelligence in Medical Care: Content Analysis of Social Media. J Med Internet Res.

